# Anaphylactic shock following intravenous ranitidine in rural Nepal: a case report

**DOI:** 10.1186/s12245-025-01101-0

**Published:** 2025-12-16

**Authors:** Rojee Shrestha, Ashal Timalsina, Arjun Gaire, Roshan Acharya, Anupa Subedi, Aayusha Suwal

**Affiliations:** Pashupati Chaulagain Memorial Hospital, Dolakha, Nepal

**Keywords:** Ranitidine, Anaphylaxis, Epinephrine, Shock

## Abstract

**Background:**

Ranitidine, a histamine-2 (H2) receptor antagonist, is widely used for acid-peptic disorders. Although generally safe, it is a rare but recognized cause of drug-induced anaphylaxis, with an estimated incidence of 0.2–0.7% for H2 receptor blockers and proton pump inhibitors. We report a near-fatal case of ranitidine-induced anaphylactic shock successfully managed in a rural hospital.

**Case presentation:**

A 35-year-old female developed sudden shortness of breath, hypotension, and drowsiness within minutes of receiving a 50 mg intravenous (IV) dose of ranitidine for epigastric discomfort at a local clinic. She had no prior exposure to ranitidine or known allergies. On arrival, her blood pressure was 60 mmHg systolic, pulse 130/min, and SpO₂ 60%. She had diffused urticaria and wheezing. A diagnosis of anaphylactic shock was made. Immediate management included high-flow oxygen, intramuscular epinephrine (0.5 mg, 1:1000), followed by intravenous hydrocortisone. Significant improvement occurred within 10 minutes and she was discharged after 24 hours of observation.

**Conclusion:**

This case highlights that ranitidine, although commonly used, can rarely trigger severe anaphylactic shock even in patients without prior exposure or known allergies. Early recognition of the reaction and timely administration of intramuscular epinephrine were key to the patient’s rapid recovery. Awareness of this potential adverse reaction is important for all clinicians who administer H2-receptor antagonists.

## Background

Ranitidine, a histamine-2 (H2) receptor antagonist, is a widely used drug for its anti-secretory properties of gastric acid, given usually for acid peptic disorders [1]. While generally considered safe, it is a rarely reported but well-documented cause of anaphylaxis. The incidence of anaphylactic reaction to H2 receptor blockers (Cimetidine and Ranitidine) and PPI (Pantoprazole, Omeprazole and lansoprazole) was together found to be 0.2 to 0.7% [2]. We report a case of near-fatal anaphylactic shock following IV ranitidine, successfully resuscitated in a rural hospital, to reinforce awareness of this adverse event and discuss the practical management challenges in such settings.

## Case presentation

### Timeline of events

0 min: 50 mg IV ranitidine administered by paramedic at local clinic.

~ 10 min: Onset of dyspnea, pruritus, generalized rash,

lightheadedness, involuntary passage of urine and stool.

~ 15 min: Arrival at district hospital emergency department.

Immediate: Diagnosis of anaphylaxis; epinephrine, oxygen, and fluids.

administered.

5 min after epinephrine: Marked hemodynamic and respiratory.

improvement.

24 h: No biphasic reaction; discharged stable.

A 35-year-old female presented to our rural district hospital with an acute onset of difficulty breathing, lethargy and an inability to stand. The patient was accompanied by the paramedic who had administered the medication at the local clinic. He confirmed that intravenous ranitidine was given and provided the used ampoule of ranitidine (50 mg/2 mL) as documentation. The medication had been administered by the paramedic as part of routine care at the clinic. The patient also had an involuntary passage of stool and urine which are the neurological manifestations of profound shock with only 1.5% cases reporting loss of bowel and bladder control [3] and no neurological deficits were found during the examination. The ranitidine preparation administered was a commercially available 50 mg/2mL formulation (Brand name: RANIVA; Batch number: RAS044 Expiry date: 06/26).

The patient had no history of chronic illnesses, no prior surgeries, and no known drug or food allergies. This was her first documented exposure to an H2 receptor antagonist (Ranitidine).

Patient’s systolic blood pressure was 60 mmHg, diastolic blood pressure was not recordable, pulse rate was 130 beats per minute, regular, respiratory rate was 32 breaths per minute, SpO2 60% on room air. The patient was oriented to time, place and person. GCS was 15/15. Widespread raised and erythematous wheals were observed without swelling of lips, face, eyelids or elsewhere. Pallor was noted. On palpation of the skin, it felt cold and clammy. Wheals were blanchable on pressure. Laboured and rapid breathing was noted with use of accessory muscles. Cyanosis and audible stridor were absent. Chest wall appeared symmetrical. On palpation, symmetrical chest expansion was present with symmetrical tactile fremitus on both sides of the chest. On percussion, normal resonance was observed at all areas of the chest. On auscultation, bilaterally decreased air entry was noted with diffuse wheeze. No crackles or stridor were heard. There were no any focal neurological deficits.

Her ECG showed sinus tachycardia (Heart rate of 140 beats per minute) with regular rhythm and no ischemic changes. Her General Random Blood Sugar was 98 mg/dl. Routine Labs were sent after stabilization: Complete Blood Count, Renal Function Tests and Liver Function Tests were all within normal limits.

A diagnosis of anaphylactic shock was made immediately based on the temporal relationship to the drug, the multi-system involvement (respiratory, cardiovascular, cutaneous) and the profound shock state as per the definition criteria of WHO ICD 11, 2019 [4].

The management followed a sequential protocol. First the patient was placed in a supine position with legs elevated. High-flow oxygen at 15 L/min was administered via a face mask with a reservoir. Two large-bore (16G) IV cannulas were secured. A rapid intravenous crystalloid fluid bolus (Normal Saline) was initiated. She received a total of 2 L of normal saline. Inj. Epinephrine 0.5 mg (0.5 mL of 1:1000 solution) was administered intramuscularly into the anterolateral thigh. Inj. Hydrocortisone 200 mg IV and Inj. Pheniramine 22.75 mg IV was also given. Within 5 min of epinephrine administration, the patient became more alert, her blood pressure improved to 130/80 mmHg, heart rate decreased to 90 bpm, and SpO2 rose to 98% on oxygen. The wheezing was resolved. Oxygen was successfully weaned off after 30 min as she maintained SpO2 > 95% on room air. She was monitored in the hospital for 24 h. She remained hemodynamically stable throughout her 24-hour observation period and was discharged the next day with complete resolution of her symptoms. She was counseled extensively regarding lifelong avoidance of ranitidine and possible cross-reactivity with other H2 receptor antagonists such as famotidine and nizatidine due to similar side chains in the ring structure, which has been documented in some immunologic reactions [5].

She was advised to use proton-pump inhibitors safely as alternative acid-suppressive therapy. She was also provided with a written allergy alert card and instructed on the importance of seeking urgent medical care in the event of recurrent allergic reactions. The patient was referred for outpatient allergy/immunology evaluation for possible ranitidine-specific IgE testing or excipient hypersensitivity assessment, although access to such testing is limited in our setting.

## Discussion

Anaphylaxis is a severe, systemic hypersensitivity reaction that is rapid in onset and characterized by life-threatening airway, breathing, and/or circulatory problems, and that is usually associated with skin and mucosal changes [6].

A study showed that most cases (57%) of anaphylaxis are due to unspecified triggers, 27% cases are associated with food and 12% of cases are medication associated [7]. The drug categories most frequently linked to anaphylaxis include antibiotics, monoclonal antibodies (mAbs), nonsteroidal anti-inflammatory drugs (NSAIDs) and acetaminophen. In contrast, the classes most commonly associated with fatal anaphylactic reactions are antibiotics, radiocontrast agents, and medications used during surgical procedures [8].

The overall mortality rate from anaphylaxis across all causes ranged from 0.002 to 2.51 deaths per million person-years. Fatal anaphylaxis triggered by food (0.002–0.29 per million person-years) was comparatively uncommon, whereas drug-induced anaphylaxis showed a higher mortality range (0.004–0.56), exceeding that associated with Hymenoptera venom (0.02–0.61). This highlights that medications are a leading contributor to fatal anaphylactic events [9].

The most commonly reported adverse effects of Ranitidine include headache, fatigue, dizziness, and mild gastrointestinal symptoms such as diarrhoea, constipation, and nausea [10]. Hypersensitivity reactions to ranitidine are uncommon; however, their clinical presentation can vary widely. Most cases involve mild to moderate skin and mucosal manifestations, while severe reactions such as bronchospasm and anaphylaxis occur only rarely [11].

H2-antihistamines are sometimes used as adjunctive therapy in the management of anaphylaxis, both with and without circulatory shock [12]. Interestingly, although H2-antihistamines help manage allergic reactions, they themselves have been implicated in rare cases of anaphylaxis, with drugs like ranitidine reported to trigger severe hypersensitivity reactions in certain individuals. Clinical manifestations of anaphylaxis result from IgE mediated direct release of histamine and other inflammatory mediators from mast cells [13].

A study in Korea revealed that among total anaphylaxis cases reported, intravenous ranitidine was seven times more implicated than oral ranitidine [14]. This is likely due to the immediate systemic exposure after an intravenous dosing. Anaphylaxis following oral ranitidine has also been reported, and all such cases were of moderate severity, requiring no cardiovascular or pulmonary.

Resuscitation whereas in contrast, intravenous administration was more often associated with severe reactions [15].

U Rethnam reported a case of ranitidine anaphylaxis in a case of acute pancreatitis [16]. In our case, the patient had no known comorbidities. However, evidence indicates that asthma, COPD and other chronic pulmonary diseases. significantly increase the risk of adverse outcomes among hospitalized patients. experiencing anaphylaxis [17].

Anaphylaxis occurring upon first exposure to ranitidine has been reported in the literature. In a study by Antonicelli et al., an 18-year-old male developed intraoperative anaphylaxis with elevated serum levels of ranitidine-specific IgE [18]. Similarly, in our case, the patient had no known previous exposure to ranitidine, further supporting the likelihood of an anaphylactic reaction occurring upon first contact with the drug.

Prompt administration of epinephrine is the cornerstone of anaphylaxis management, as early treatment significantly improves patient outcomes and helps prevent progression to severe or fatal reactions. Conversely, delayed use or failure to administer epinephrine has been strongly associated with increased mortality [19]. Multiple studies have demonstrated that epinephrine is often underutilized in the management of anaphylaxis and this underuse is primarily attributed to a lack of timely recognition of anaphylaxis, limited knowledge regarding the appropriate indications for epinephrine administration and insufficient confidence in its use [20, 21]. These challenges are likely to be even more pronounced in low-resource settings, where limited training opportunities may further hinder prompt and effective treatment. In our case, epinephrine was administered promptly, followed by corticosteroids, as evidence suggests that corticosteroid therapy may help prevent or shorten the duration of protracted or biphasic anaphylactic reactions [22].

Some ranitidine injection solution contains preservatives such as phenol However; studies have shown that patients with ranitidine anaphylaxis often do not cross react with other H2 receptor antagonist such as cimetidine or famotidine or to proton pump inhibitors [14]. This lack of cross reactivity further supports that the unique chemical structure of ranitidine is the cause of anaphylaxis.

## Conclusion

This case highlights an important lesson for healthcare providers in low-resource settings about the need for a high vigilance for suspicion for anaphylaxis, even when administering commonly used medications. It underscores the importance of ensuring that life-saving drugs such as epinephrine are readily available at all times and that medical personnel are adequately trained to promptly recognize and manage anaphylactic reactions.

## Data Availability

No datasets were generated or analyzed during the current study.

## Abbreviations

H2: Histamine 2
mg: milligrams
mmHg: millimeters of mercury
PPI: Proton Pump Inhibitor
Iv: Intravenous
ER: Emergency Room
GCS: Glasgow coma scale
GRBS: General Random Blood Sugar
dl: deciliter
L: Liter
ml: milliliters
Inj: Injection
IV: Intravenous
